# Characterization of a mutant *samhd1* zebrafish model implicates dysregulation of cholesterol biosynthesis in Aicardi-Goutières syndrome

**DOI:** 10.3389/fimmu.2023.1100967

**Published:** 2023-03-06

**Authors:** Sarah E. Withers, Charlie F. Rowlands, Victor S. Tapia, Frances Hedley, Ioana-Emilia Mosneag, Siobhan Crilly, Gillian I. Rice, Andrew P. Badrock, Andrew Hayes, Stuart M. Allan, Tracy A. Briggs, Paul R. Kasher

**Affiliations:** ^1^ Division of Neuroscience, School of Biological Sciences, Faculty of Biology, Medicine and Health, Manchester Academic Health Science Centre, The University of Manchester, Manchester, United Kingdom; ^2^ Geoffrey Jefferson Brain Research Centre, The Manchester Academic Health Science Centre, Northern Care Alliance National Health Service (NHS) Foundation Trust, The University of Manchester, Manchester, United Kingdom; ^3^ Division of Evolution, Infection and Genomic Sciences, School of Biological Sciences, Faculty of Biology, Medicine and Health, University of Manchester, Manchester, United Kingdom; ^4^ Manchester Centre for Genomic Medicine, St. Mary’s Hospital, Manchester University National Health Service (NHS) Foundation Trust, Manchester Academic Health Science Centre, Manchester, United Kingdom; ^5^ Medical Research Council (MRC) Human Genetics Unit, University of Edinburgh, Edinburgh, United Kingdom; ^6^ Genomic Technologies Core Facility, Faculty of Biology, Medicine and Health, The University of Manchester, Manchester, United Kingdom; ^7^ Lydia Becker Institute of Immunology and Inflammation, Faculty of Biology, Medicine and Health, Manchester Academic Health Science Centre, University of Manchester, Manchester, United Kingdom

**Keywords:** Aicardi Goutières syndrome, cholesterol, zebrafish disease models, type I interferonopathy, SAMHD1

## Abstract

Aicardi-Goutières syndrome (AGS1-9) is a genetically determined encephalopathy that falls under the type I interferonopathy disease class, characterized by excessive type I interferon (IFN-I) activity, coupled with upregulation of IFN-stimulated genes (ISGs), which can be explained by the vital role these proteins play in self-non-self-discrimination. To date, few mouse models fully replicate the vast clinical phenotypes observed in AGS patients. Therefore, we investigated the use of zebrafish as an alternative species for generating a clinically relevant model of AGS. Using CRISPR-cas9 technology, we generated a stable mutant zebrafish line recapitulating AGS5, which arises from recessive mutations in *SAMHD1*. The resulting homozygous mutant zebrafish larvae possess a number of neurological phenotypes, exemplified by variable, but increased expression of several ISGs in the head region, a significant increase in brain cell death, microcephaly and locomotion deficits. A link between IFN-I signaling and cholesterol biosynthesis has been highlighted by others, but not previously implicated in the type I interferonopathies. Through assessment of neurovascular integrity and qPCR analysis we identified a significant dysregulation of cholesterol biosynthesis in the zebrafish model. Furthermore, dysregulation of cholesterol biosynthesis gene expression was also observed through RNA sequencing analysis of AGS patient whole blood. From this novel finding, we hypothesize that cholesterol dysregulation may play a role in AGS disease pathophysiology. Further experimentation will lend critical insight into the molecular pathophysiology of AGS and the potential links involving aberrant type I IFN signaling and cholesterol dysregulation.

## Introduction

Aicardi-Goutières syndrome (AGS) is a rare, type I interferonopathy that primarily presents as a severe childhood-onset neurological disease. To date, nine genetic subtypes have been identified that can be caused by mutations in *TREX1* (AGS1), the three non-allelic components of RNASEH2 (*RNASEH2A-C*; AGS2-4), *SAMHD1* (AGS5), *ADAR1* (AGS6), *IFIH1* (AGS7), *LSM11* (AGS8) or *RNU7-1* (AGS9) (1–6). Mutations in these genes lead to abnormal processing, sensing or metabolism of self-nucleic acids and subsequent activation of type I interferon (IFN-I) signaling and enhanced expression of interferon stimulated genes (ISGs). AGS patients exhibit a spectrum of neurological (and non-neurological) phenotypes, which can vary in severity, and most frequently include brain atrophy, intracranial calcification, microcephaly, white matter lesions, motor dysfunction and intellectual disability (7–9). Given the significant auto-inflammatory component of the disease, these phenotypes are thought to manifest due to a neurotoxic consequence of excessive IFN-I signaling, but to date this hypothesis remains unproven. Furthermore, AGS symptoms can present in a heterogeneous fashion, with additional neurological phenotypes observed in specific genetic subtypes, including *ADAR1*-related bilateral striatal necrosis (10, 11), *ADAR1, IFIH1* and *RNASEH2B*-related spastic paraplegia (12) and *SAMHD1*-related cerebrovasculopathy and stroke (13–16).

Given AGS is a rare disease, the availability of patient-derived material for experimental investigation is limited. As such, animal models of AGS are utilized to help study disease mechanisms and for pre-clinical drug discovery. However, to date, few animal models of AGS successfully recapitulate both the inflammatory and neurological phenotypes associated with the disease (17). Previously, we have shown that morpholino-mediated knockdown of *samhd1* in zebrafish embryos recapitulates aspects of the IFN-I and stroke phenotypes observed in AGS5 (18). In the present study, we expand on this work and characterize a novel CRISPR-Cas9 induced stable *samhd1* mutant zebrafish line. The aim of this work was to further demonstrate the translational utility of zebrafish disease modelling for studying AGS and to make novel mechanistic observations associated with the disease.

## Materials and methods

### Zebrafish husbandry

Adult zebrafish husbandry was approved by The University of Manchester Animal Welfare and Ethical Review Board, and all experiments were performed in accordance with U.K Home Office regulations (PPL: P132EB6D7). The zebrafish used in this study were raised and maintained at The University of Manchester Biological Services Unit, under standard conditions, with adults housed in mixed sex tanks with a recirculating water supply maintained at 28°C under a 14/10 hour light/dark cycle, as previously described (19). Fertilized eggs were collected following natural spawning, and incubated at 28°C in fresh E3 medium. The embryos were staged according to standard guidelines (20). After termination of the experiment, all embryos were killed prior to protected status [5 days post fertilization (dpf)] using a lethal dose of Tricaine Methanesulfonate (MS222) and freezing at -20°C.

### Generation of samhd1^Δ23^ mutant line

A guide RNA (gRNA) with no predicted off-target sequences was designed for exon 4 of *samhd1* using CHOPCHOP (21). gRNA incorporating this target sequence was generated from a polymerase chain reaction (PCR) amplification (Forward primer: 5’- TAATACGACTCACTATAGGCGTCACATTAAGCAGCTCGGGTTTTAGAGC-3’; Reverse primer: 5’-AAAAGCACCGACTCGGTGCCACTTTTTCAAG-3’) including the remaining sequence of *S. pyogenes* chimeric single gRNA through *in vitro* transcription using a HiScribe T7 Quick kit (New England Biolabs). Precipitation of the gRNA and synthesis of Cas9 RNA was performed as previously described (22). The resulting RNAs were mixed with 0.05% phenol red, 120 mM KCl and 20mM HEPES, ph7.0, and ~1 nl of the mix was injected into the yolk of wild type (WT) AB fertilized eggs at the one cell stage to produce F0 crispants. To identify founders, F0 adults were individually crossed with wild type (WT) AB animals and the resulting embryos were assessed for indels using Hyp188I restriction analysis. This process identified a founder animal harboring a 23bp deletion in exon 4 (*samhd1* ^Δ23^) that was predicted to lead to a frameshift. Following confirmation of germline transmission, the F0 *samhd1* ^Δ23^ was crossed again with WT AB fish to produce an F1 generation. Following F1 heterozygous mutant incrosses, we ascertained that homozygotes (*samhd1* ^Δ23/Δ23^) were viable and capable of breeding. Subsequent generations were genotyped following PCR and Sanger sequencing of fin clip genomic DNA, using primers that flanked the 23bp deletion (forward primer 5’-GTGTTTAATGACCCCATCCA-3’; reverse primer: 5’-CCTATGGAGTGCTCAAATCG-3’).

To generate the amino acid sequence, the zebrafish WT samhd1 transcript was obtained from Ensembl (23) and inputted into ExPASy for translation (24). For the *s*amhd1^Δ23/Δ23^ sequence, the 23 bp deletion determined by Sanger sequencing was removed from the WT transcript before translation. For this study, all experiments were performed on an F4/F5 generation and comparisons were made between age matched WT and homozygous mutants.

### Quantitative PCR

Total RNA was extracted from pooled groups of whole larvae (n=20) or dissected larval heads (n=30) at 5 days post-fertilization (dpf) using a standard TRIzol (Invitrogen) method. Complementary DNA (cDNA) was synthesized from 800ng RNA as previously described (18). Quantitative PCR (qPCR) was performed on a StepOne Plus Real Time PCR machine (Applied Biosystems). To assess *samhd1* expression in whole larvae, cDNA was combined with power SYBR green mastermix (Applied Biosystems) and primers (Eurofins Genomics) that flanked the 23bp deletion (Forward: 5’-AGAACATCATCTGCCGCCGG-3’; Reverse: 5’-CCAGTTCCTTCGCCCAGTCC-3’). Primers targeting the housekeeping gene *hprt1* were used to normalize expression (Forward: 5’-GGACTTCATCCTCAAGAG-3’; Reverse: 5’-GTTCTAGCAGCGTCTTCATCG-3’). To assess expression of ISGs (*isg15* (18)*, isg12* (Dr03140917_g1) and *stat1b* (Dr03151121_m1)) and the cholesterol biosynthesis gene *hmgcrb* (Dr03128326_m1) in dissected larval heads, a Taqman (Life Technologies) protocol was performed, as previously described (18).

### Head size measurements

Anaesthetized WT and *samhd1* ^Δ23/Δ23^ embryos were fixed overnight in 4% paraformaldehyde (PFA; Alfa Aesar) at 2 dpf before washing in 1% PBS-Tritonx. To image the samples, 80% glycerol was added to the embryos which were placed into a nunc glass base dish 12 mm. The samples were individually imaged in a ventral orientation using a Leica M165FC light stereo microscope with DFC7000T camera, and processed using LAS-X v3.3.3.16958 software (Leica). To assess for microcephaly, the distance between the eyes was measured alongside full length of the embryo (μm). The head/body ratio was determined from these two values, and normalized to the average WT head/body ratio, to produce a microcephaly index (ratio). Measurements were obtained using Image J (version 1.52a). n=8 embryos per group, for each biological replicate, repeated 3 independent times.

### TUNEL staining

At 2 dpf, PFA fixed WT and *samhd1* ^Δ23/Δ23^ embryos were stained using the Click- iT Plus TUNEL assay kit (Invitrogen) following the manufacturer guidelines. For imaging, samples were mounted in a lateral position in 80% glycerol on a nunc glass base dish 12 mm (Thermo Fisher) and imaged on a Leica M205 FA Stereo fluorescence microscope using a 5x/0.50 PlanAPO LWD objective, captured using a DFC 365FX camera and processed using LAS AF v3.1.0.8587 software (Leica). Images were analyzed using the manual cell counting software on Image J (version 1.52a). Analyses were performed on n= 6-8 embryos from 3 independent replicates.

### Locomotion assay

Swimming was measured from 3 – 5 dpf in WT and *samhd1* ^Δ23/Δ23^ larvae using DanioVision camera chamber and ethovision XT software (Noldus, version 11), as previously described (25). Cumulative duration of movement was measured in 3 independent replicates, with n=22-24 larvae per replicate.

### Atorvastatin treatment and o-dianisidine treatment

To assess for brain hemorrhages, WT and *samhd1* ^Δ23/Δ23^ embryos (n=20 per group) were treated with atorvastatin (ATV; Merck) at 32 hours post-fertilization (hpf), as described previously (25). To visualize bleeds, ATV treated embryos were stained from 54 hpf using an o-dianisidine (Sigma) protocol (26). The percentage of embryos with hemorrhages within each treatment/genotype group was determined from 3 independent replicates.

### Acquisition of AGS patient whole blood samples

Whole blood from AGS patients with confirmed mutations in *TREX1*, *RNASEH2A*, *RNASEH2B*, *RNASEH2C*, *SAMHD1*, *ADAR1* or *IFIH1* was taken to allow for whole genome RNA sequencing, as described previously (27, 28). In addition, samples from non-AGS patient age-matched controls were obtained. Briefly, AGS patients were identified through either direct clinical contact with the appropriate physicians, or through a referral process. The use of patient materials has been approved from the Leeds (East) research ethics committee (reference number: 07/Q1206/7) and South Centre – Hampshire A research ethics committee (reference number 17/SC/0026), whilst consent was also obtained from parents of the affected patients.

### AGS patient whole blood RNA sequencing

The Paxgene (PreAnalytix) whole blood RNA extraction method for RNA sequencing has been described previously, and the AGS patient RNA sequencing data-sets have been published elsewhere (27–29). RNA concentration was assessed with a spectrophotometer (FLUOstar Omega, Labtech), and 1 μg of mRNA from each sample was diluted into 20 μl nuclease-free water. Subsequently, the quality and integrity of the RNA samples were assessed using a 2200 TapeStation (Agilent Technologies). A poly-A enrichment library was then generated using the TruSeq^®^ Stranded mRNA assay (Illumina), according to the manufacturer’s protocol, and 76 bp (+ indices) paired-end sequencing carried out on an Illumina HiSeq 4000. Raw sequencing output was demultiplexed (allowing one mismatch) and the Binary Base Call (BCL) sequence file format was converted to the text-based sequencing data file format (FASTQ) using Illumina’s bcl2fastq software, version 2.17.1.14. Low quality bases and adaptor sequences were trimmed using Trimmomatic, and reads aligned to the Genome Reference Consortium Human Build 37 (GRCh37) genome using the two-pass mode of the Spliced Transcripts Alignment to a Reference (STAR) aligner (v2.5.3a), as well as to the transcriptome according to the GENCODE v19 human gene annotation (downloaded from https://www.gencodegenes.org/human/release_19.html). For each patient sample, the RNA-Seq by Expectation Maximization (RSEM) software package (v1.3.0) was used to calculate gene expression values, in transcripts per million (TPM). Subsequently, fold change was determined from TPM values, and then Log transformed (Log2).

### Statistics

All data were analyzed using GraphPad Prism 8.1.2 (GraphPad software Inc). All zebrafish experiments, except from ATV treatment were analyzed using either an unpaired t test, or Mann Whitney test, depending on normality, established by the Shapiro Wilks normality test. ATV treatment was analyzed using a two-way ANOVA with sidak’s multiple comparisons test. AGS patient RNA seq data was analyzed using a one-way ANOVA with Dunnetts multiple comparisons test, comparing each AGS gene with the control group.

## Results

### Generation of the samhd1^Δ23^ line

**Figure 1:**
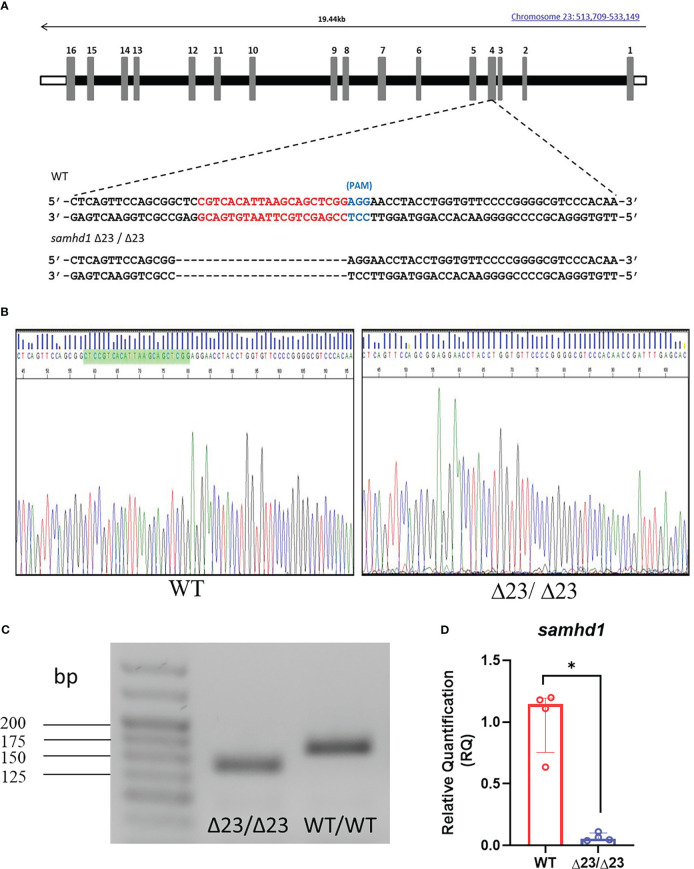
Generation of the *samhd1* ^Δ23/Δ23^ line. **(A)** gRNA sequence (red) designed to target a site within exon 4 of the *samhd1* gene to initiate a 23 bp deletion in injected embryos. **(B)** Sanger sequencing of WT and *samhd1* ^Δ23/Δ23^ mutants from fish derived from F1 generation incross. Green highlighted area on WT sequencing represents the site of the 23 bp deletion, causing a frameshift on the mutant sequence. **(C)** Amplification of *samhd1* transcripts from mutant and WT zebrafish embryos using exon 4 specific primers, yielded a single PCR product with clearly visible reduction in band size, as a result of 23 bp deletion. Length of *samhd1* exon 4 is 161 bp in WT embryos, with the mutants producing a band of 138 bp. **(D)** qPCR analysis of *samhd1* gene expression in 5 dpf WT and *samhd1* ^Δ23/Δ23^ larvae. Data analyzed using Mann Whitney U test and presented as median ± IQR (*P=0.0286). n=20 larvae per group, per biological replicate, repeated 4 times.

Following on from the *samhd1* MO model, which successfully recapitulated aspects of the AGS5 phenotype, a stable mutant zebrafish line was subsequently generated to facilitate long term studies (18). To generate a stable samhd1 loss of function model, a gRNA was designed using the CHOPCHOP software, targeting exon 4 of the *samhd1* gene (**Figure 1A**). A 23 bp deletion was confirmed by Sanger sequencing and PCR in the F1 generation (**Figures 1B, C**). To determine whether this mutation resulted in loss of transcript, RNA was harvested from WT and *samhd1* ^Δ23/Δ23^ larvae at 5 dpf for qPCR analysis of the *samhd1* gene, where the mutants exhibited almost a complete loss in *samhd1* expression compared to WT larvae (P<0.05) (**Figure 1D**). Translation analysis of genomic sequences highlighted that the *samhd1* ^Δ23^ mutation introduces a premature stop codon prior to the HD domain which is predicted to produce a truncated amino acid sequence (**Supplementary Figure 1**).

### Loss of samhd1 induces AGS-like phenotypes in zebrafish larvae

**Figure 2:**
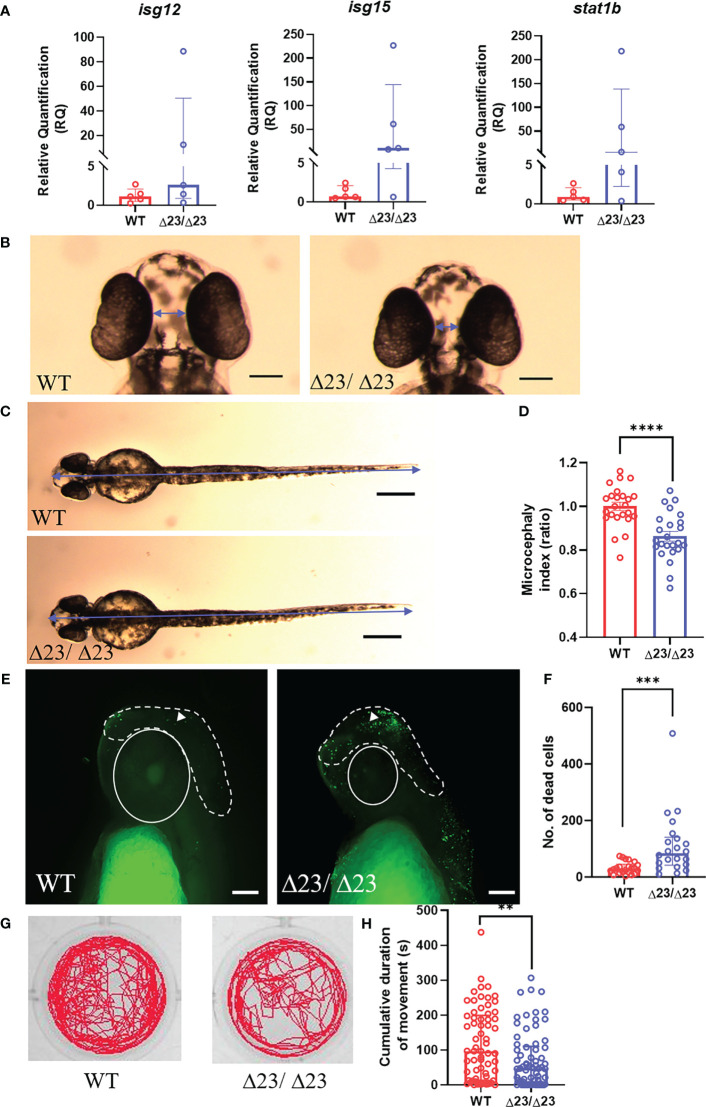
*samhd1* ^Δ23/Δ23^ zebrafish exhibit neurological phenotypes. **(A)** Specific ISG expression (from taqman probes for *isg12*, *isg15* and *stat1b*, normalized to the housekeeper gene *hprt1*) measured in 5 dpf WT and samhd1^Δ23/Δ23^ larval heads, n=30 per biological replicate, repeated 5 times. **(B-D)** Microcephaly phenotype identified by ventral measurements between the eyes **(B)** and full body length **(C)** (blue arrows) to generate a microcephaly index (ratio) determined by distance between the eyes and body length **(D)**. Data analyzed using unpaired t test (****P<0.0001) presented as mean ± SEM. **(E, F)** Enhanced head cell death in *samhd1* ^Δ23/Δ23^ embryos determined through TUNEL staining. White dotted line indicates area of interest for manual cell counting, excluding the eyes, mouth and ears. White triangle denotes TUNEL positive cell used for analysis. n=6-8 embryos per group, with 3 biological replicates. Scale bar= 100 μm. Quantification of number of dead cells **(F)**. Data analyzed using Mann Whitney U test (***P<0.001) presented as median ± IQR. **(G)** Representative examples of locomotor tracks for 4 dpf samhd1^Δ23/Δ23^ and WT larvae. **(H)** Significant decrease in cumulative duration of movement at 4 dpf. n=22-24 larvae per group, with three biological replicates. Data analyzed using a Mann Whitney U test (**P<0.001).

One of the primary hematological hallmarks of AGS is the excessive upregulation of IFN-I and the subsequent ISG signature, which is often used as a clinical diagnostic tool (7, 27, 30). Therefore, in an attempt to phenocopy this observation, we measured ISG expression in larval heads (**Figure 2A**). Variable expression of *isg12*, *isg15* and *stat1b* was observed in *samhd1* ^Δ23/Δ23^ larvae compared to the WTs. However, although variable, the *samhd1* ^Δ23/Δ23^ larvae appeared to display increased expression of each ISG, although this did not reach statistical significance. Expression analysis of other ISGs displayed similar variability (data not shown).

Severe neurological manifestations are another important clinical feature of AGS, with patients presenting with a range of CNS symptoms (7, 31). To characterize the initiation of neurological phenotypes in the zebrafish model, we first measured head size, as microcephaly is a common clinical manifestation of AGS (32–34). The distance between the eyes after ventral imaging of fixed embryos at 2 dpf was used to measure microcephaly (**Figure 2B**). Full body length was also measured to determine a head to body length ratio, also referred to as a microcephaly index, normalized to the mean of the WT group (**Figure 2C**). The microcephaly index was significantly reduced by 15% in *samhd1* ^Δ23/Δ23^ embryos (P<0.0001), thus indicating that loss of samhd1 is associated with smaller head size in zebrafish (**Figure 2D**).

Previous studies have suggested that microcephaly can be attributed to an increase in neuronal cell death (35). In an attempt to assess this, TUNEL staining was used to identify apoptotic cells in the head region. Manual counting of apoptotic cells in the indicated region (**Figure 2E**) revealed a significant increase in brain cell death in the *samhd1* ^Δ23/Δ23^ embryos (P<0.001) (**Figure 2F**).

Subsequently, we aimed to establish whether the microcephaly and increased brain cell death in *samhd1* ^Δ23/Δ23^ embryos also contributed to functional defects. From a clinical perspective, a wide range of motor disabilities are observed across all human AGS subtypes (7). Swimming behavior can be readily assessed in zebrafish larvae as a readout for motor dysfunction, as observed in other zebrafish models of disease (25). We tracked swimming behavior in the larvae between 3 - 5 dpf. No difference in movement was observed at either 3 dpf or 5 dpf (data not shown), however, a ~50% reduction in cumulative duration of movement was observed in the larvae at 4dpf (P<0.01), implying a transient physical deficit exists in the *samhd1* ^Δ23/Δ23^ model (**Figures 2G, H**).

### samhd1^Δ23/Δ23^ zebrafish exhibit cerebral hemorrhages associated with cholesterol dysregulation

We next tested for AGS5-related cerebrovasculopathy, as the *samhd1* MO model developed spontaneous cerebral hemorrhages (18). At baseline conditions, only a small proportion (5%) of *samhd1* ^Δ23/Δ23^ embryos developed cerebral hemorrhages, whilst no hemorrhages were observed in the WT embryos.

**Figure 3:**
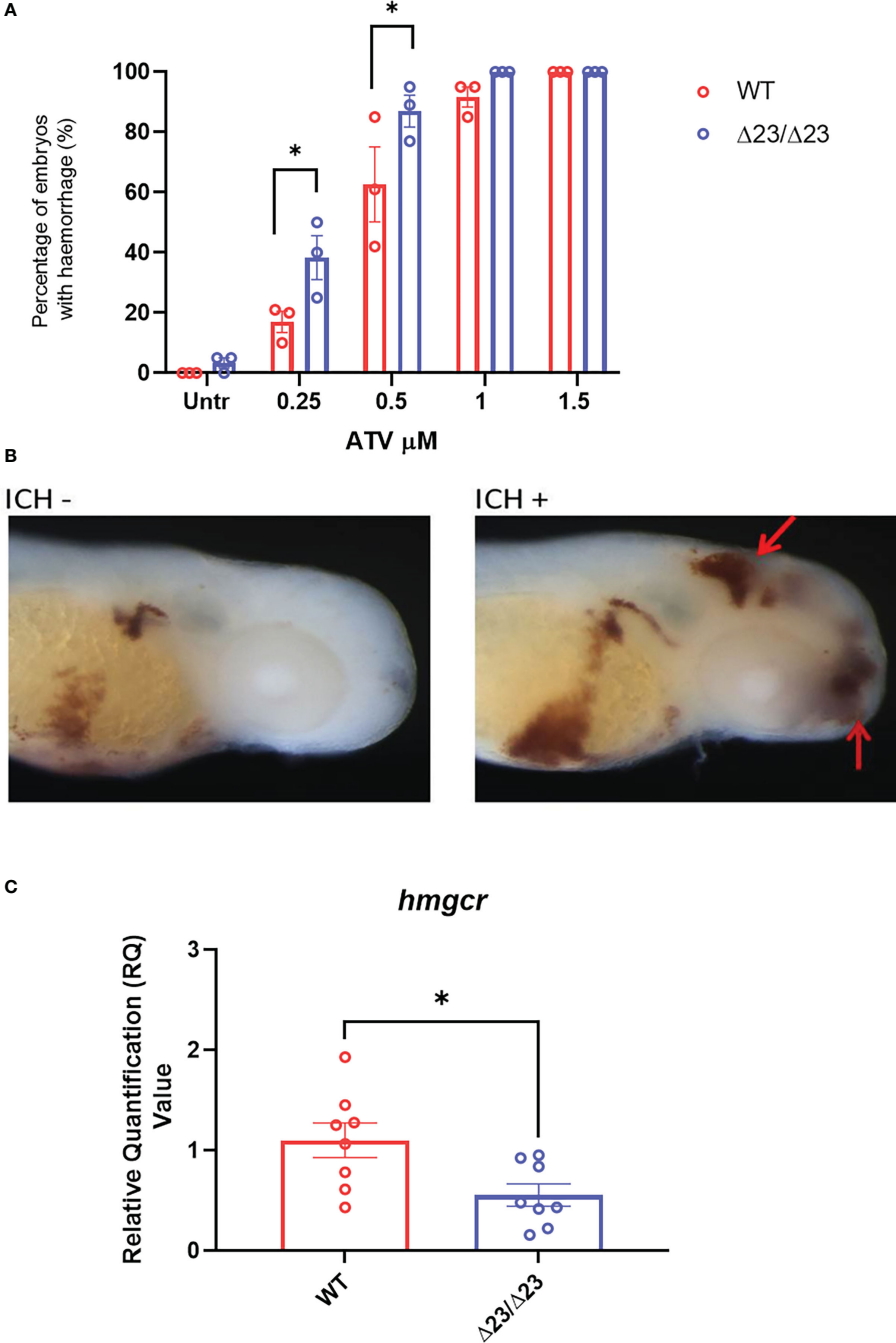
*samhd1* ^Δ23/Δ23^ zebrafish exhibit cerebrovascular defects associated with cholesterol dysregulation. **(A)** WT and *samhd1* ^Δ23/Δ23^ embryos were treated with increasing concentrations (0.25-1.5 μM) of ATV added *via* water bath incubation and scored on the presence or absence of blood in the brain. N=20 embryos treated per group, with the percentage of animals per group which developed hemorrhages determined, repeated three times. Data analyzed using a two-way ANOVA with sidak’s multiple comparisons test (*P<0.05). **(B)** Representative images of embryos stained for o-dianisodine with and without brain hemorrhage, denoted by red arrow. **(C)** qPCR analysis of *hmgcr* expression in the WT and mutant larvae heads at 5 dpf. Data analyzed using unpaired t test (*P<0.05) and presented as mean ± SEM.

Pharmacological inhibition of the 3-hydroxy-3-methylglutaryl-CoA reductase (hmgcr) pathway, using statins, has previously been shown to induce brain specific hemorrhages in zebrafish (25, 26). The hemorrhages arise due to a reduction in *de novo* cholesterol biosynthesis, resulting in lowered cholesterol essential for neurovascular integrity (26). As only a small percentage of embryos spontaneously hemorrhaged in the *samhd1* ^Δ23/Δ23^ model at baseline, we tested whether the mutants may be more susceptible to brain hemorrhage following exposure to low concentrations of ATV. Following water bath incubation at ~30 hpf, ATV-induced hemorrhages were consistently identified from ~52 hpf. The incidence of hemorrhages increased in a dose-dependent manner for both mutant and WT groups, however, there was a significant increase in cerebral hemorrhage frequencies in the *samhd1* ^Δ23/Δ23^ embryos at the lower doses of 0.25 μM (P<0.05) and 0.5 μM (P<0.05) ATV: 17% versus 38% and 63% versus 87% respectively (**Figures 3A, B**). We postulated that increased susceptibility to ATV-induced cerebral hemorrhages may indicate a deficiency in *hmgcr* expression in the homozygotes. Therefore, we measured *hmgcr* transcript by qPCR in larval heads and identified a ~50% reduction in *samhd1* ^Δ23/Δ23^ larvae compared to WT (**Figure 3C**). Together, these data suggest that the *samhd1* ^Δ23/Δ23^ embryos possess a genetically-induced susceptibility to cerebral hemorrhages that is associated with dysregulated cholesterol biosynthesis.

### RNA sequencing reveals dysregulation of cholesterol biosynthesis gene expression in AGS patient whole blood

**Figure 4:**
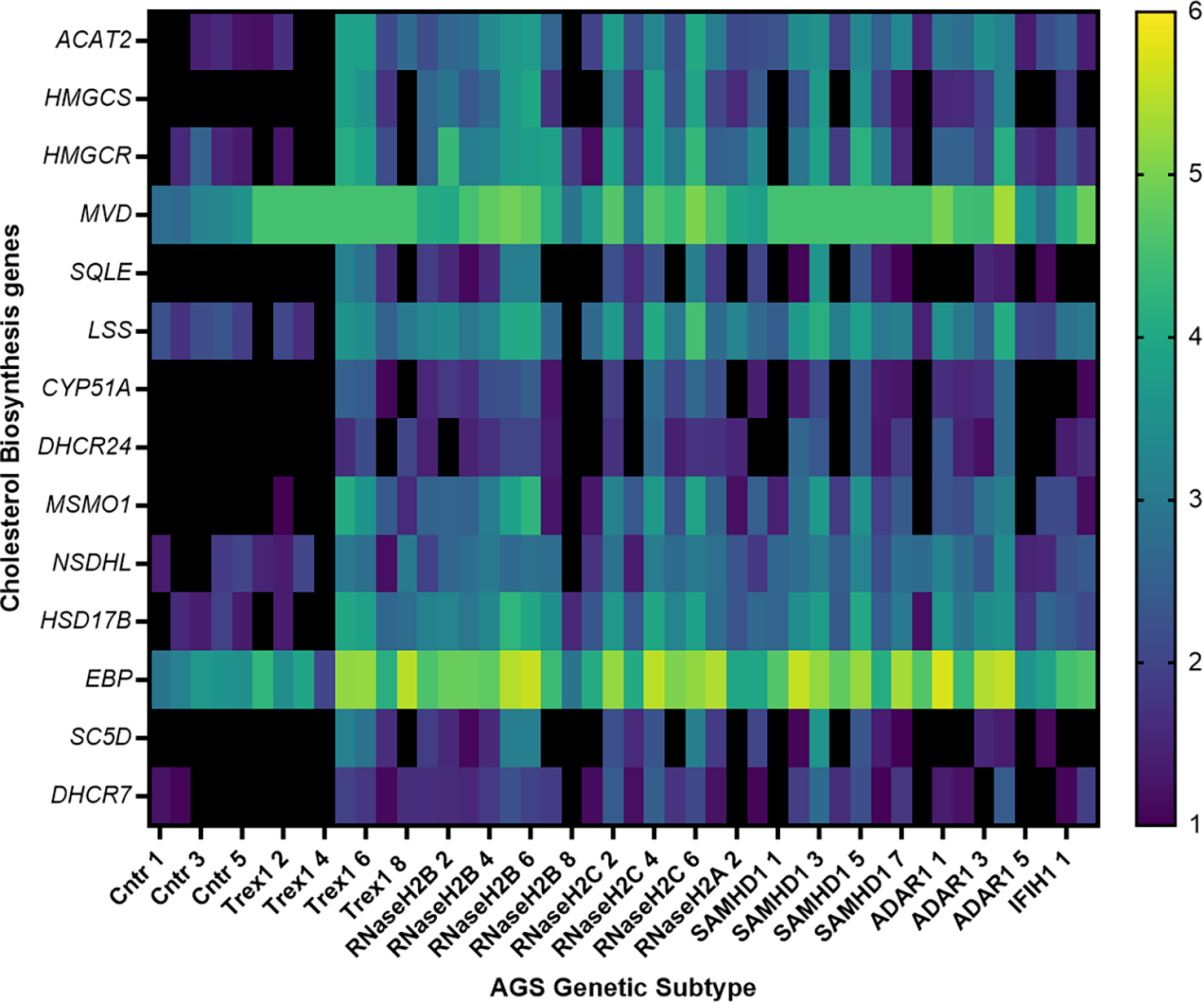
Transcriptional dysregulation of cholesterol biosynthesis genes in AGS patient blood. RNA from AGS1-7 patient whole blood was analyzed using the RSEM software to determine the TPM of each cholesterol biosynthesis gene for the AGS patients. Data was then normalized to an age-matched control group, and underwent a log2 transformation. The heat map scale on right hand side of figure indicates color changes representing 1-6 fold changes in expression. Sample sizes for each AGS subtype: Control n=5, TREX1 n=8, RNaseH2B n=8, RNaseH2C n=6, RNaseH2A n= 3, SAMHD1 n=8, ADAR1 n=6 and IFIH1 n=2. Data analyzed using a one-way ANOVA with Dunnetts multiple comparison test, comparing each AGS gene with the control group.

AGS5 patients frequently present with cerebrovascular disease (13–16). Furthermore, reductions in cholesterol have been associated with increased risk of hemorrhagic stroke in the general population (36–40). As such, we hypothesized that a reduction in cholesterol biosynthesis gene expression could be specifically associated with AGS5 and not observed across other AGS subtypes in which cerebrovasculopathy is not reported. To test this hypothesis and to validate the translational relevance of the *samhd1* ^Δ23/Δ23^ zebrafish observation, we assessed the expression of several genes encoding enzymes involved in the multiple arms of the cholesterol biosynthesis pathway (**Supplementary Figure 2**) in existing RNA sequencing data obtained from AGS1-7 patient whole blood samples. This analysis revealed an overall increase in the expression of 9 of the 14 cholesterol biosynthesis genes measured (*ACAT2, MVD, CYP51A, DHCR24, MSMO1, NSDHL, HSD17B, EBP, SC5D*), compared to an age matched control group (**Figure 4**). This pattern was found across all AGS subtypes, excluding AGS1 patients, where no differences were observed. Although directionally inverse to the zebrafish model, these observations implicate defective cholesterol biosynthesis in the pathophysiology of AGS.

## Discussion

Here, we provide further evidence that zebrafish disease modelling represents a useful *in vivo* tool for studying AGS5, building upon the knowledge gained from the *samhd1* MO zebrafish model previously described by our group (18). Furthermore, this work has provided new insight into AGS pathophysiology by implicating a potential role for defective cholesterol biosynthesis in the *samhd1* ^Δ23/Δ23^ zebrafish and patient samples.

Loss of *samhd1* produced a number of clinically relevant phenotypes in the zebrafish model, Firstly, the ISG response in larval heads was largely variable, and as such, we were unable to confirm a consistent ISG phenotype. However, high expression levels of ISGs were observed in a proportion of mutant larvae suggesting the discrepancy we observe may be attributed to distinct intra-larval variation. Indeed, heterogeneous ISG responses and symptom severity is also observed in the human condition (41). Intra-larval variation may be apparent due to the non-sterile aquatic environment the adult fish are housed in. Indeed, Rutherford and colleagues have previously suggested zebrafish are subjected to additional inflammagen exposure and other challenges from the water, compared to a sterile mouse environment (17). Upregulation of ISGs has been previously identified in a number of AGS animal models, resulting in multi-organ inflammation. However, the brain has remained largely unaffected, until relatively recently with the advent of newer murine models for *Rnaseh2b* and *Adar1* mutations, and now the *samhd1^Δ23/Δ23^* zebrafish model described here (42–45).

Due to the variation in ISG expression, we were unable to confirm that this upregulation is solely accountable for the presence of other observed neurological phenotypes, such as microcephaly, enhanced brain cell death and the locomotor deficits that were observed in the mutants. Future studies will assess the effects of IFN inhibition on these outcomes in this model. Interestingly, DNA damage, rather than IFN signaling has recently been hypothesized to be the cause of neural dysfunction in a neuro-progenitor conditional *Rnaseh2b* ^-/-^ mouse model (42, 46). Further study is required to determine whether DNA damage is the primary cause of these phenotypes in the zebrafish model.

The *samhd1* MO model gave rise to a spontaneous brain hemorrhage phenotype, providing one of the first examples of a sub-type specific phenotype identified in a pre-clinical AGS model (18). For AGS5 this includes cerebral large artery disease, manifesting as stenosis, aneurysms, moyamoya, ischemic and hemorrhagic stroke (7, 13–15). As such, it has been hypothesized these cerebrovascular phenotypes reveal an as yet uncharacterized function of the SAMHD1 protein, relating to vascular homeostasis (14, 18). Moreover, the cerebrovascular defects found in AGS5 may be attributed to the spatial expression profile of SAMHD1. Whole tissue microarray from human donors identified constitutive expression of SAMHD1 within the vascular endothelium, including the CNS. However, expression profiles should also be established for the other AGS causative genes (47).

Whilst a small proportion of *samhd1* ^Δ23/Δ23^ embryos did hemorrhage under baseline conditions, this was at a largely reduced frequency than observed in the previous *samhd1* morphants (18). Therefore, we hypothesized that the stable mutants may possess a more subtle defect within the cerebrovasculature, which in isolation is insufficient to cause spontaneous hemorrhages, but may instead lead to an increased propensity to cerebral bleeds. To test this hypothesis, embryos were treated with a range of doses of the hemorrhage inducing drug ATV, where we observed a significant increase in hemorrhaging rates at the lowest doses in *samhd1* ^Δ23/Δ23^ fish (25, 26). Given that ATV exerts its effects by targeting the cholesterol biosynthesis rate limiting enzyme, *hmgcr*, and inhibiting downstream cholesterol and lipid biosynthesis, this suggested the mutants may possess a baseline deficiency within the cholesterol biosynthesis pathway. Gene expression analysis of *hmgcr* highlighted a significant reduction in the *samhd1* mutants compared to the WTs, thus providing novel evidence that a loss of *samhd1* results in a genetic defect in cholesterol biosynthesis. Indeed, the relationship between increased cerebral hemorrhage rates and inhibition of cholesterol synthesis aligns with the clinical observation that reduced cholesterol levels are associated with increased risk of hemorrhagic stroke (36–40). Therefore, we hypothesized that this observation may account specifically for the cerebrovascular disease observed in AGS5 patients.

Patient whole blood RNA-seq analysis revealed the cholesterol biosynthesis gene dysregulation was not specific for SAMHD1-related AGS, indicating this observation cannot be directly attributed to the AGS5-related cerebrovasculopathy, as comparable effects were observed within AGS2-4 and 6-7 genetic subtypes. We propose that cholesterol biosynthesis dysregulation may be more broadly associated with aberrant IFN-I signaling, as highlighted by others (48). Notably, there were directional differences in dysregulated cholesterol gene expression identified between the *samhd1* ^Δ23/Δ23^ larvae (downregulation) and patients (upregulation). We postulate these differences may reflect acute versus chronic exposure to aberrant IFN-I signaling, whereby a compensatory effect is initiated to counteract the inhibition of cholesterol synthesis over time. Such compensation is more likely feasible in patients over time, in comparison to the first five days of life in zebrafish. Moreover, we have only focused on cholesterol biosynthesis gene expression within the patients and zebrafish model, which does not fully portray the complex relationship between the antiviral response, initiated by IFN-I, and regulation of cholesterol.

As such, further research is required to understand this potential relationship between IFN-I signaling and cholesterol biosynthesis dysregulation specifically in AGS patients. We speculate that such a defect might contribute to certain pathological features of AGS, including white matter loss. The brain is cholesterol rich and a significant proportion is contained within myelin. Therefore, under disease conditions, defects in cholesterol may dysregulate myelin formation and lead to white matter degeneration. Indeed, in a mouse model of cerebrovascular disease, a high cholesterol diet was associated with white matter defects and cognitive decline (49).

Moreover, we appreciate that peripheral measurements of cholesterol biosynthesis gene expression do not directly represent the cholesterol lipid levels within the brain and CNS, and as such, future experimentation would involve taking plasma and CSF cholesterol measurements, whilst also investigating pathways other than cholesterol biosynthesis, such as cholesterol efflux. Whilst material is scarce, an interesting avenue for future studies would be to focus on assessing brain cholesterol levels in AGS post mortem tissue.

Irrespective of our focus on the periphery, previous literature has identified direct correlative relationships between plasma cholesterol levels and a number of different brain pathologies, including stroke (both ischemic and hemorrhagic), intracranial calcifications, and chronic neurodegenerative disorders (50, 51). Together, this demonstrates the importance of this RNA sequencing result in an attempt to understand aspects of the encephalopathic nature of AGS.

A limitation of the current study is that potential off-target effects initiated by the sgRNAs in zebrafish have not been fully assessed. Future studies could focus on using next generation sequencing to identify potential off-target candidates to further validate this model. Nevertheless, confirmation of cholesterol biosynthesis defects in both zebrafish and AGS patients provides confidence that this particular observation is likely *bona fide*.

To conclude, we have generated a stable mutant zebrafish model of AGS5, which exhibited several clinically relevant phenotypes, including neurological manifestations which have rarely been replicated in other pre-clinical models of AGS, thus reinforcing the usefulness of zebrafish as a pre-clinical species for type I interferonopathy research. The zebrafish model highlighted a potential novel association with AGS and dysregulation in cholesterol biosynthesis, which will spark further investigation into the role of cholesterol dysregulation in disease progression within AGS, whilst also increasing our understanding of the complexities surrounding IFN-I signaling and cholesterol homeostasis.

## Data availability statement

Publicly available datasets were analyzed in this study. This data can be found here: https://www.ebi.ac.uk/biostudies/arrayexpress/studies/E-MTAB-9722?accession=E-MTAB-9722 and https://www.ebi.ac.uk/biostudies/arrayexpress/studies/E-MTAB-7087?accession=E-MTAB-7087.

## Ethics statement

The studies involving human participants were reviewed and approved by Leeds (East) research ethics committee (reference number: 07/Q1206/7) South Centre-Hampshire A research ethics committee (reference number 17/SC/0026). Written informed consent to participate in this study was provided by the participants’ legal guardian/next of kin. The animal study was reviewed and approved by The University of Manchester Animal Welfare and Ethical Review Board.

## Author contributions

SW designed and performed experiments, analyzed the data, and wrote the manuscript. GR, VT, FH and I-EM designed and performed experiments. CR and AH performed data analysis. AB and SC provided technical expertise. SA and TB provided supervision and revised the manuscript. PK supervised the study and wrote the manuscript. All authors contributed to the article and approved the submitted version.
