# Supplementary material for: Characterization of a mutant samhd1 zebrafish model implicates dysregulation of cholesterol biosynthesis in Aicardi-Goutières syndrome
Source: Front Immunol. 2023 Mar 6;14:1100967. doi: 10.3389/fimmu.2023.1100967 (PMC10025490; doi:10.3389/fimmu.2023.1100967)
Supplement: Supplementary file 1 [file Presentation_1.pptx]

## Slide 1
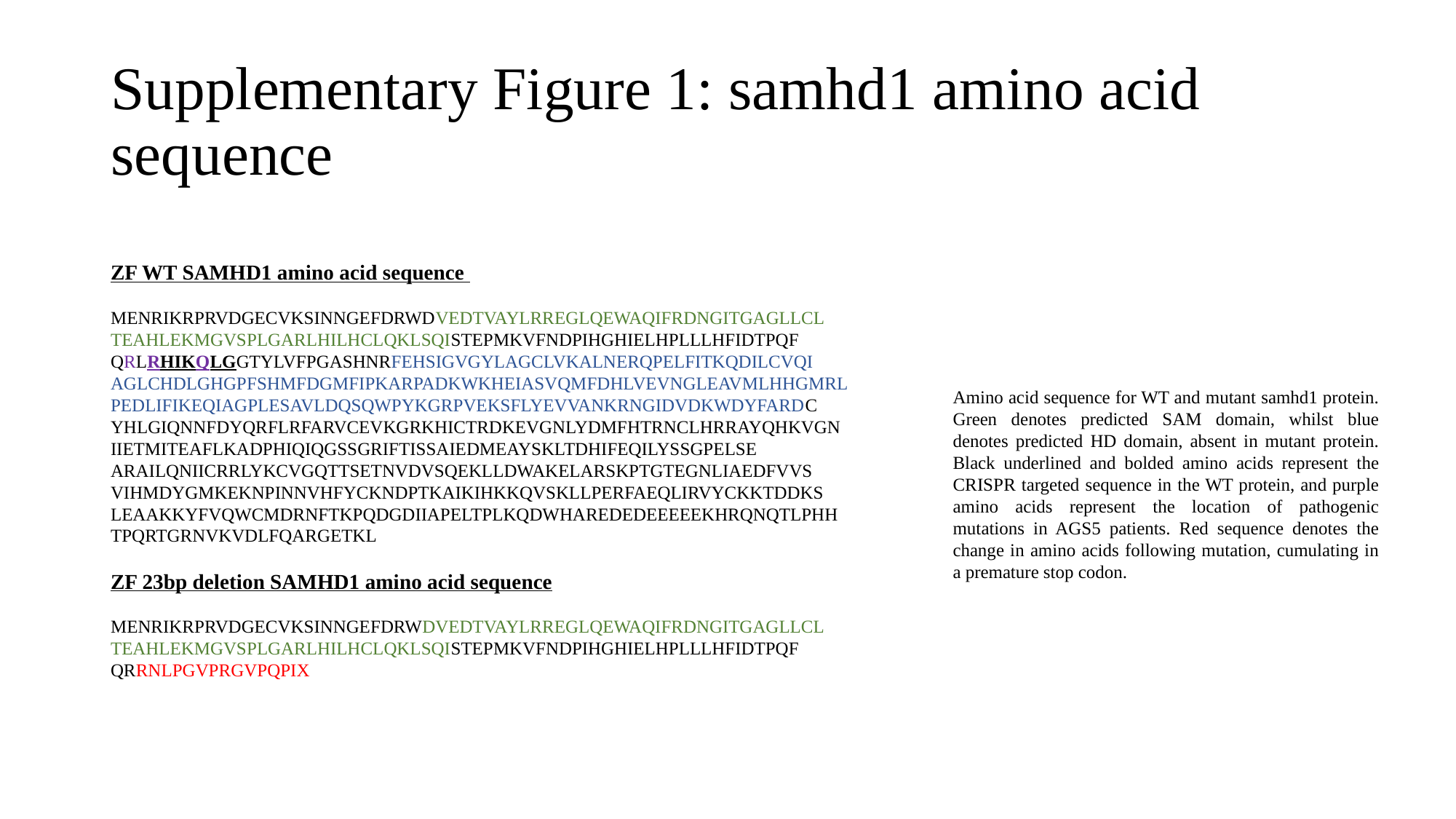

# Supplementary Figure 1: samhd1 amino acid sequence
ZF WT SAMHD1 amino acid sequence
MENRIKRPRVDGECVKSINNGEFDRWDVEDTVAYLRREGLQEWAQIFRDNGITGAGLLCL
TEAHLEKMGVSPLGARLHILHCLQKLSQISTEPMKVFNDPIHGHIELHPLLLHFIDTPQF
QRLRHIKQLGGTYLVFPGASHNRFEHSIGVGYLAGCLVKALNERQPELFITKQDILCVQI
AGLCHDLGHGPFSHMFDGMFIPKARPADKWKHEIASVQMFDHLVEVNGLEAVMLHHGMRL
PEDLIFIKEQIAGPLESAVLDQSQWPYKGRPVEKSFLYEVVANKRNGIDVDKWDYFARDC
YHLGIQNNFDYQRFLRFARVCEVKGRKHICTRDKEVGNLYDMFHTRNCLHRRAYQHKVGN
IIETMITEAFLKADPHIQIQGSSGRIFTISSAIEDMEAYSKLTDHIFEQILYSSGPELSE
ARAILQNIICRRLYKCVGQTTSETNVDVSQEKLLDWAKELARSKPTGTEGNLIAEDFVVS
VIHMDYGMKEKNPINNVHFYCKNDPTKAIKIHKKQVSKLLPERFAEQLIRVYCKKTDDKS
LEAAKKYFVQWCMDRNFTKPQDGDIIAPELTPLKQDWHAREDEDEEEEEKHRQNQTLPHH
TPQRTGRNVKVDLFQARGETKL
ZF 23bp deletion SAMHD1 amino acid sequence
MENRIKRPRVDGECVKSINNGEFDRWDVEDTVAYLRREGLQEWAQIFRDNGITGAGLLCL
TEAHLEKMGVSPLGARLHILHCLQKLSQISTEPMKVFNDPIHGHIELHPLLLHFIDTPQF
QRRNLPGVPRGVPQPIX
Amino acid sequence for WT and mutant samhd1 protein. Green denotes predicted SAM domain, whilst blue denotes predicted HD domain, absent in mutant protein. Black underlined and bolded amino acids represent the CRISPR targeted sequence in the WT protein, and purple amino acids represent the location of pathogenic mutations in AGS5 patients. Red sequence denotes the change in amino acids following mutation, cumulating in a premature stop codon.

## Slide 2
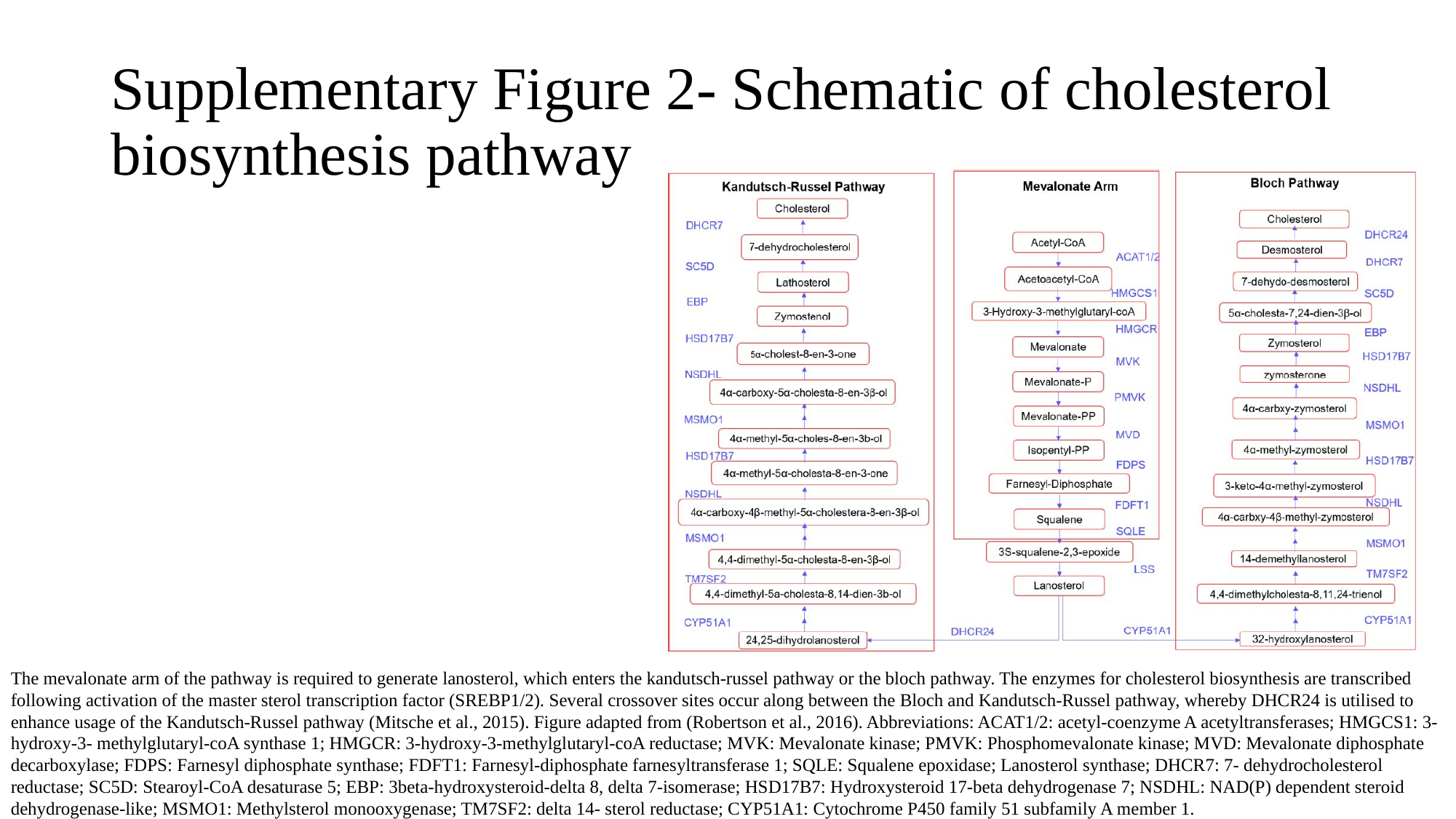

# Supplementary Figure 2- Schematic of cholesterol biosynthesis pathway
The mevalonate arm of the pathway is required to generate lanosterol, which enters the kandutsch-russel pathway or the bloch pathway. The enzymes for cholesterol biosynthesis are transcribed following activation of the master sterol transcription factor (SREBP1/2). Several crossover sites occur along between the Bloch and Kandutsch-Russel pathway, whereby DHCR24 is utilised to enhance usage of the Kandutsch-Russel pathway (Mitsche et al., 2015). Figure adapted from (Robertson et al., 2016). Abbreviations: ACAT1/2: acetyl-coenzyme A acetyltransferases; HMGCS1: 3-hydroxy-3- methylglutaryl-coA synthase 1; HMGCR: 3-hydroxy-3-methylglutaryl-coA reductase; MVK: Mevalonate kinase; PMVK: Phosphomevalonate kinase; MVD: Mevalonate diphosphate decarboxylase; FDPS: Farnesyl diphosphate synthase; FDFT1: Farnesyl-diphosphate farnesyltransferase 1; SQLE: Squalene epoxidase; Lanosterol synthase; DHCR7: 7- dehydrocholesterol reductase; SC5D: Stearoyl-CoA desaturase 5; EBP: 3beta-hydroxysteroid-delta 8, delta 7-isomerase; HSD17B7: Hydroxysteroid 17-beta dehydrogenase 7; NSDHL: NAD(P) dependent steroid dehydrogenase-like; MSMO1: Methylsterol monooxygenase; TM7SF2: delta 14- sterol reductase; CYP51A1: Cytochrome P450 family 51 subfamily A member 1.

## Slide 3
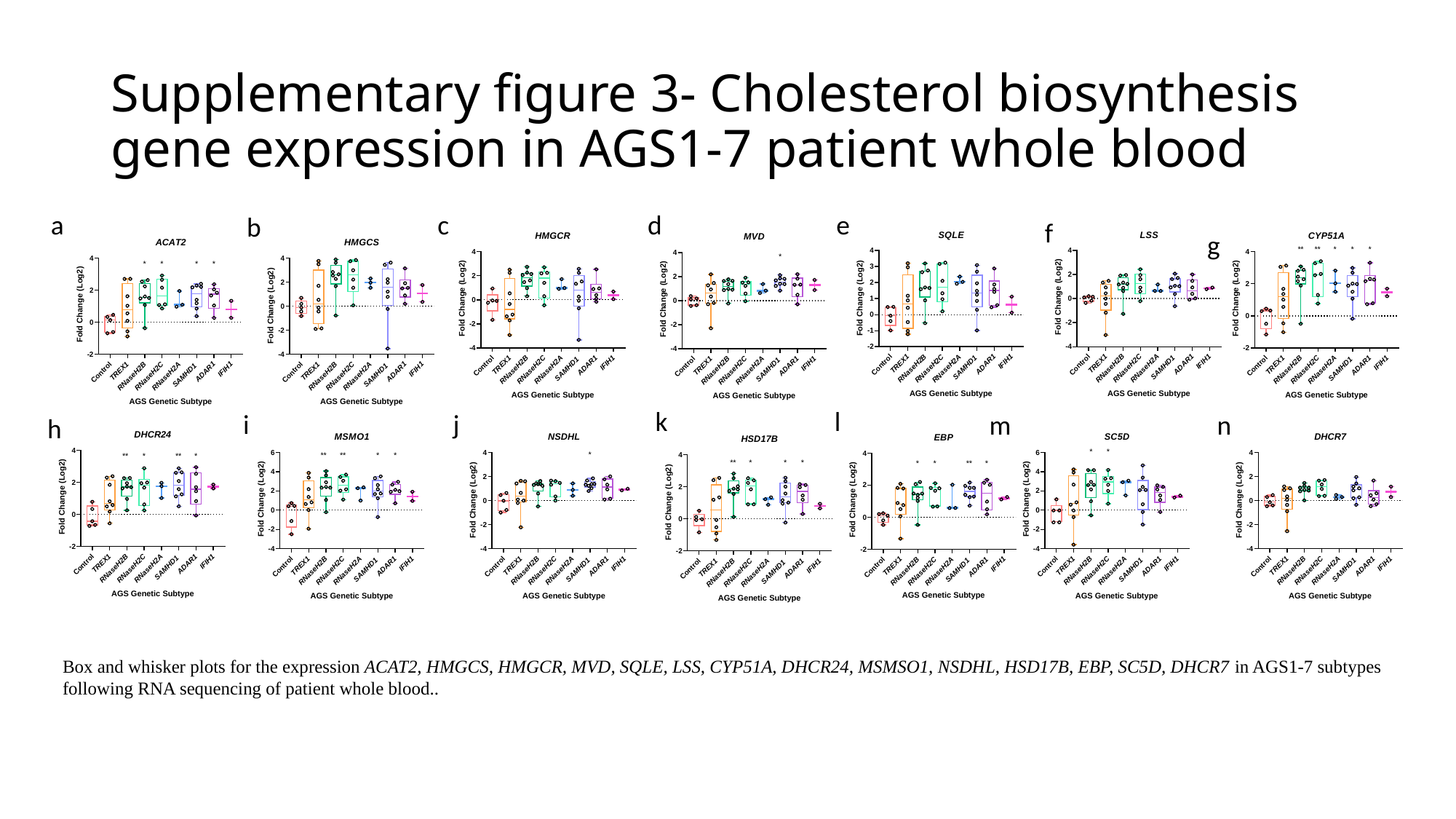

# Supplementary figure 3- Cholesterol biosynthesis gene expression in AGS1-7 patient whole blood
a
c
d
e
b
f
g
k
l
i
j
m
n
h
Box and whisker plots for the expression ACAT2, HMGCS, HMGCR, MVD, SQLE, LSS, CYP51A, DHCR24, MSMSO1, NSDHL, HSD17B, EBP, SC5D, DHCR7 in AGS1-7 subtypes following RNA sequencing of patient whole blood..

## Slide 4
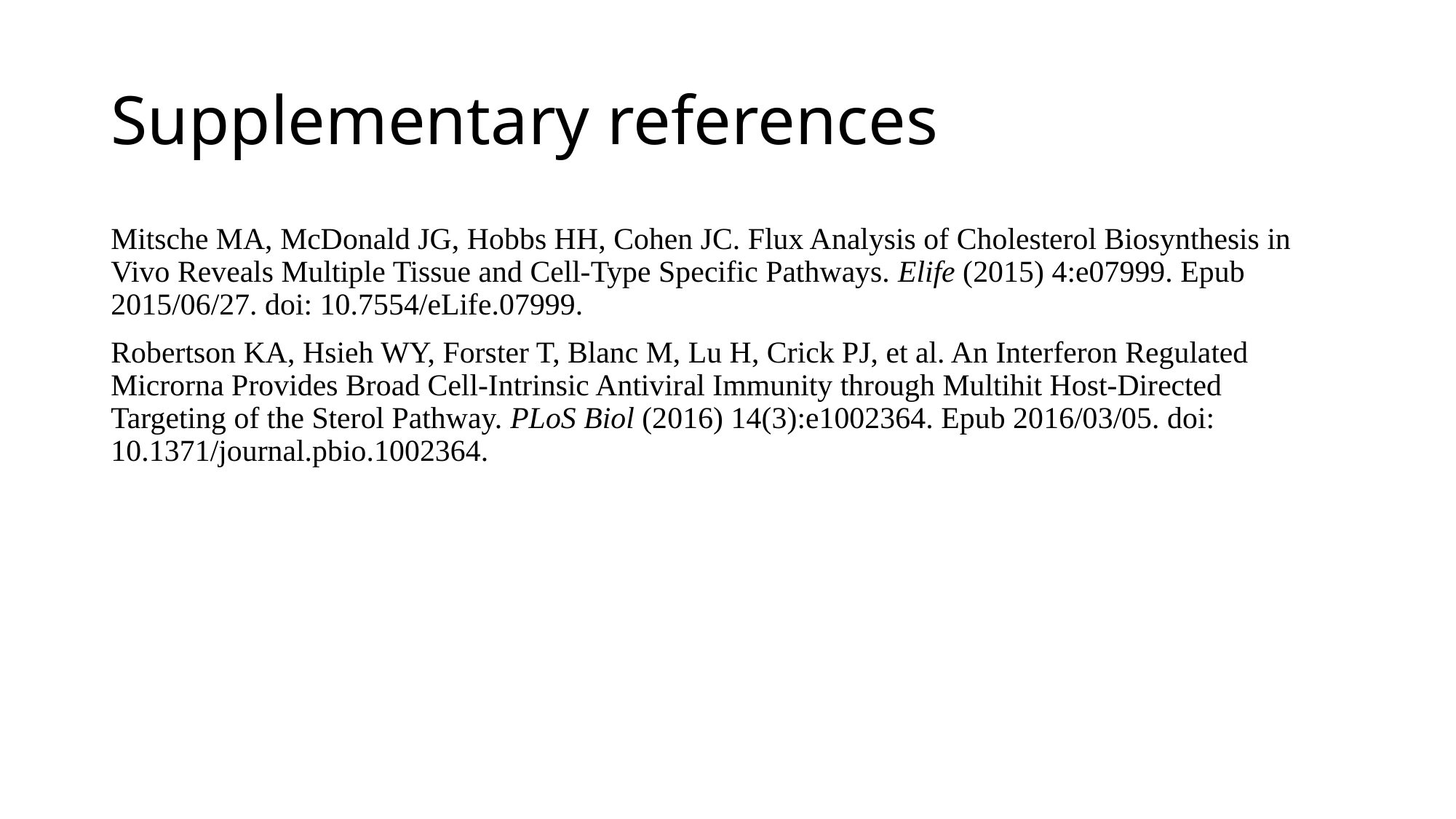

# Supplementary references
Mitsche MA, McDonald JG, Hobbs HH, Cohen JC. Flux Analysis of Cholesterol Biosynthesis in Vivo Reveals Multiple Tissue and Cell-Type Specific Pathways. Elife (2015) 4:e07999. Epub 2015/06/27. doi: 10.7554/eLife.07999.
Robertson KA, Hsieh WY, Forster T, Blanc M, Lu H, Crick PJ, et al. An Interferon Regulated Microrna Provides Broad Cell-Intrinsic Antiviral Immunity through Multihit Host-Directed Targeting of the Sterol Pathway. PLoS Biol (2016) 14(3):e1002364. Epub 2016/03/05. doi: 10.1371/journal.pbio.1002364.
